# Discordant cerebrospinal fluid and positron emission tomography amyloid biomarkers in an *APP* mutation carrier presenting corticobasal syndrome

**DOI:** 10.1002/alz.70823

**Published:** 2025-10-21

**Authors:** Feng‐Tao Liu, Xin‐Yi Li, Jia‐Ying Lu, Chuan‐Tao Zuo

**Affiliations:** ^1^ Department of Neurology National Clinical Research Center for Aging and Medicine & National Center for Neurological Disorders Huashan Hospital Fudan University Shanghai China; ^2^ Department of Nuclear Medicine & PET Center Huashan Hospital Fudan University Shanghai China

**Keywords:** ^18^F‐florbetapir, Alzheimer's disease, amyloid, Aβ structures, biomarkers, cerebrospinal fluid, positron emission tomography imaging

## Abstract

**INTRODUCTION:**

While amyloid cerebrospinal fluid (CSF) and positron emission tomography (PET) biomarkers are considered interchangeable indicators of Alzheimer's disease (AD) pathology, biomarker discrepancies can occur but remain poorly characterized.

**METHODS:**

We evaluated ^18^F‐florbetapir amyloid PET, ^18^F‐Florzolotau PET (tau pathology), magnetic resonance imaging (MRI) findings, and CSF biomarkers in a 59‐year‐old man carrying the pathogenic *APP* p.K687Q mutation, who presented with possible corticobasal syndrome.

**RESULTS:**

CSF analysis revealed reduced amyloid beta (Aβ)_1‐42_ (503.44 pg/mL) and Aβ_1‐42_/Aβ_1‐40_ ratio (0.044), indicating amyloid pathology. Conversely, ^18^F‐florbetapir PET was visually negative (standardized uptake value ratio [SUVR] 0.97; −11.8 Centiloids). ^18^F‐Florzolotau PET demonstrated AD‐typical tau deposition, whereas MRI revealed extensive white matter hyperintensities, enlarged perivascular spaces, and a temporal microbleed.

**DISCUSSION:**

The observed discordance suggests that CSF and PET amyloid biomarkers can diverge in certain patients. Potential mechanisms include polymorphic Aβ fibrils lacking ^18^F‐florbetapir binding sites, excess non‐fibrillar aggregates, low fibril density, or contributions from cerebral amyloid angiopathy.

**Highlights:**

- CSF Aβ and ^18^F‐florbetapir PET findings showed a mismatch in a patient with an APP mutation.
- Amyloid pathology should not be excluded despite negative ^18^F‐florbetapir PET findings.
- Mismatch may reflect altered ligand binding or fibril structural variants.
- Comorbid cerebral amyloid angiopathy may contribute to biomarker discrepancies.

## 1 INTRODUCTION

The diagnostic landscape of Alzheimer's disease (AD) has been fundamentally reshaped by the incorporation of biomarkers into both clinical and research settings. Within the amyloid/tau/neurodegeneration (ATX[N]) framework, positron emission tomography (PET), cerebrospinal fluid (CSF) analysis, and, more recently, blood‐based markers have become central to defining AD pathology and guiding differential diagnosis across neurodegenerative syndromes. Amyloid PET imaging and CSF amyloid beta (Aβ) peptide concentrations are presently designated as Core 1 biomarkers of early AD pathology.^1^ Given their high concordance across studies,^2^ current diagnostic criteria treat CSF and PET as interchangeable measures of cerebral Aβ deposition. However, emerging evidence suggests that these modalities can occasionally yield discordant results – a phenomenon that remains poorly understood and inadequately documented in the literature.

To illustrate this diagnostic challenge, we describe a patient who exhibited a striking divergence between CSF and amyloid PET biomarkers. The patient carried a pathogenic amyloid precursor protein (*APP*) mutation and presented with possible corticobasal syndrome (CBS). CSF analysis demonstrated reduced Aβ_1‐42_ levels together with a decreased Aβ_1‐42_/Aβ_1‐40_ ratio, whereas amyloid PET with ^18^F‐florbetapir showed no evidence of cortical amyloid deposition. This case underscores the importance of comprehensive multimodal biomarker assessment and highlights potential mechanisms underlying discrepancies between CSF and PET measures of amyloid pathology.

## 2 METHODS AND RESULTS

**FIGURE 1:**
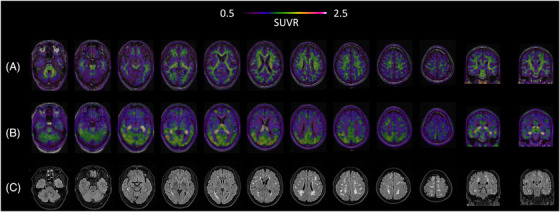
Brain imaging findings in a patient carrying p.K687Q *APP* mutation. (A) ^18^F‐florbetapir amyloid PET showing negative results. (B) ^18^F‐Florzolotau PET demonstrating asymmetric cortical uptake (SUVR maps overlaid on structural MRI). (C) T2‐FLAIR MRI. MRI, magnetic resonance imaging; PET, positron emission tomography; SUVR, standardized uptake value ratio.

A 59‐year‐old man carrying a pathogenic *APP* mutation (p.K687Q)^3^ presented with a 3‐year history of progressive memory decline coupled with left limb stiffness, myoclonus, and hypoesthesia – resulting in a clinical diagnosis of possible CBS.^4^ CSF analysis revealed AD‐like pathology, with decreased Aβ_1‐42_ levels of 503.44 pg/mL (normal reference range: ≥888.1 pg/mL) and a reduced Aβ_1‐42_/Aβ_1‐40_ ratio of 0.044 (normal reference range: ≥0.068). Notably, ^18^F‐florbetapir amyloid PET imaging (acquisition window: 50 to 70 min after injection) – visually assessed as negative by in‐house experts^5^ – showed a global cortical standardized uptake value ratio (SUVR) of 0.97 (Centiloid scale = −11.8, reference region: whole cerebellum)^6^ (Figure 1A). By contrast, ^18^F‐Florzolotau PET (acquisition window: 90 to 110 min after injection)^7^ demonstrated asymmetric tau deposition in AD‐characteristic regions (temporal lobes, precuneus, posterior cingulate cortex) as well as in the primary motor cortex – a hallmark of CBS (Figure 1B). Complementary magnetic resonance imaging (MRI) revealed multifocal white matter hyperintensities (WMHs) and severe centrum semiovale‐enlarged perivascular spaces on T2‐weighted and T2‐Fliud Attenuated Inversion Recovery sequences, while susceptibility‐weighted imaging detected a single microbleed in the temporal lobe (Figure 1C; Video S1).

## 3 DISCUSSION

This case demonstrates a clinically significant mismatch between CSF Aβ biomarker assessments and ^18^F‐florbetapir PET imaging results in a patient with an *APP* mutation presenting with CBS. These findings underscore the importance of applying multimodal biomarker strategies judiciously when establishing a diagnosis of AD or related disorders. Importantly, our observations are consistent with previous studies describing PET‐CSF discrepancies in Aβ assessment in clinical practice.^8^, ^9^, ^10^ To account for this discordance, several mechanisms may be considered, either individually or in combination.

**RESEARCH IN CONTEXT**

1. **Systematic review**: According to AD criteria, PET and CSF biomarkers are considered interchangeable for assessing amyloid pathology; however, discrepancies remain underreported.
2. **Interpretation**: We describe a patient with a pathogenic *APP* mutation who underwent ^18^F‐florbetapir PET, ^18^F‐Florzolotau PET (tau pathology), and CSF assessments. Despite negative ^18^F‐florbetapir PET findings, CSF analysis revealed reduced Aβ_1‐42_ levels (503.44 pg/mL) and Aβ_1‐42_/Aβ_1‐40_ ratio (0.044). ^18^F‐Florzolotau PET showed AD‐characteristic uptake, whereas MRI demonstrated WMHs, enlarged perivascular spaces, and a temporal lobe microbleed.
3. **Future directions**: CSF and PET amyloid biomarkers may rarely diverge due to altered fibril morphology, non‐fibrillar aggregate formation, low density of mature fibrils, or coexisting cerebral amyloid angiopathy. These findings underscore the need for multimodal biomarker integration, tracer design informed by fibril structures, and careful clinical interpretation to better understand atypical AD biomarker signatures.

The first explanation involves tracer‐specific binding characteristics. The binding affinity of amyloid PET tracers critically depends on plaque morphology and the availability of high‐affinity binding sites. Consequently, tracers such as ^11^C‐labeled Pittsburgh Compound‐B (^11^C‐PiB) and ^18^F‐florbetapir demonstrate preferential binding to cored and neuritic plaques but show markedly lower affinity for diffuse plaques.^11^ The phenomenon of tracer selectivity was previously illustrated by Schöll et al.,^8^ who described two carriers of the Arctic *APP* mutation with reduced CSF Aβ_1‐42_ concentrations and evidence of glucose hypometabolism on ^18^F‐FDG PET in regions typically affected in AD, despite a complete absence of cortical retention on ^11^C‐PiB PET. In contrast, a carrier of the Swedish *APP* mutation demonstrated pronounced cortical uptake. The authors attributed these divergent findings to structural differences in Aβ plaque morphology. Specifically, Arctic mutation carriers were found to form ring‐shaped Aβ aggregates lacking classic β‐pleated sheet fibrils, a feature that may underlie the absence of tracer retention.

Building upon these morphological insights, recent advances in cryo‐electron microscopy (cryo‐EM) have revealed the remarkable heterogeneity of Aβ fibril structures. Specifically, Zhao and colleagues^12^ identified a novel *ex vivo* Aβ42 fibril conformation (type III) in sporadic AD, which lacked the binding channel typically present in type I Aβ42 fibrils, thereby limiting ^18^F‐florbetapir affinity. Importantly, specific genetic factors, such as the Arctic mutation, as well as chromosomal conditions like trisomy 21 (Down syndrome), have been associated with the formation of atypical Aβ fibril conformations that differ from the canonical type I and type II structures.^13^, ^14^ Although type III fibrils have not yet been directly linked to clinical PET findings, the pathogenic *APP* variant reported here (p.K687Q), located within the Aβ core, may similarly alter fibril architecture and reduce tracer binding. However, this explanation remains speculative and warrants further investigation.

A second potential mechanism relates to the density‐dependent nature of PET detection. Common PET amyloid tracers require a sufficient density of fibrillar deposits to yield detectable cortical binding.^11^ Intriguingly, certain *APP* mutations appear to favor the formation of oligomers rather than fully fibrillar aggregates.^15^, ^16^ For instance, the Osaka *APP* mutation accelerates Aβ oligomerization while simultaneously impeding fibril formation.^15^ Consistent with this mechanism, carriers of the Osaka mutation exhibited substantial tau pathology and positive ^11^C‐PBB3 tau PET signals, yet demonstrated minimal cortical binding of ^11^C‐PiB.^9^, ^10^ We hypothesize that similar mechanisms may be operative in our case.

The third explanation may involve the potential contribution of comorbidities – in particular, cerebral amyloid angiopathy (CAA). Evidence suggests that patients with CAA may exhibit reduced CSF Aβ_1‐42_ levels despite negative amyloid PET imaging findings,^17^ since vascular amyloid deposits in CAA do not significantly contribute to cortical PET signal^18^ – a distinction that may be clinically useful for differentiating CAA from sporadic AD.^19^ In our patient, MRI evidence of multispot WMHs, severe centrum semiovale perivascular spaces, and a single microbleed in the temporal lobe is consistent with concurrent possible CAA pathology (Boston criteria, version 2.0)^20^ – which could reasonably contribute to the dissociation between CSF Aβ peptide biomarkers and amyloid PET findings.

Our findings highlight an important clinical consideration in rare cases where a negative Aβ PET result does not exclude AD pathology, particularly in the presence of abnormal CSF Aβ concentrations. Critically, our case demonstrates that heterogeneous Aβ structures and other contributing factors can evade detection by current PET tracers, potentially leading to false‐negative findings. Although our data are limited by the single case presentation and require validation in larger cohorts of both sporadic and familial patients, this rare case underscores the need for multimodal biomarker integration in atypical AD presentations and supports the development of next‐generation tracer design informed by fibril structures.

## 4 CONCLUSION

We conclude that the exclusion of AD diagnosis should not rely solely on a negative ^18^F‐florbetapir PET scan – particularly when there is strong clinical suspicion and supporting evidence of altered ligand binding. In our patient, several factors may account for the observed discordance between CSF Aβ peptide concentrations and ^18^F‐florbetapir PET findings, including differences in Aβ plaque morphology and density, variability in tracer binding sites, structural heterogeneity of Aβ fibrils, and the presence of concurrent CAA. Collectively, these observations underscore the importance of integrating diverse biomarker data in clinical evaluation and the critical role of expert interpretation. Furthermore, advancing structural insights into tracer–fibril interactions may guide the development of improved tracers and strengthen the interpretive value of amyloid PET imaging in clinical practice.

## CONFLICTS OF INTERESTS STATEMENT

The authors declare no conflicts of interest. Author disclosures are available in the Supporting Information.

## CONSENT STATEMENT

The patient provided written consent for the publication of this case report.

## Supporting information



Supporting Information

Supporting Information
